# Supplementary material for: Identifying pragmatic solutions to reduce cigarette smoking prevalence in Indigenous North Americans: A sequential exploratory mixed-methods study protocol
Source: PLoS One. 2024 Nov 11;19(11):e0306512. doi: 10.1371/journal.pone.0306512 (PMC11554222; doi:10.1371/journal.pone.0306512)
Supplement: S1 Appendix — (DOCX) [file pone.0306512.s001.docx]

S1 Appendix: IRB approved consent documents organized by population.

Mayo Clinic: Office for Human Research Protection

**Oral Consent Script – American Indian and Alaska Native Subjects**

**Protocol Title:** Understanding barriers to smoking cessation in American Indian and Alaska Native People

**IRB #:** 23-011852

**Principal Investigator:** Dr. Ann M. Rusk, M.D., and colleagues

Please read the oral consent script below to learn more about the study.

You are being asked to participate in a research study about the experience of quitting cigarette smoking. The purpose of this research is to understand barriers to long-term smoking cessation in American Indian or Alaska Native people and barriers to quitting during pregnancy. If you self-identify as American Indian or Alaska Native and you used to or currently smoke cigarettes, you may be eligible for this study.

If you agree to participate, you will participate in either a group interview/talking circle including 3-4 people in-person, or a one-on-one discussion via phone or video call with Dr. Rusk and/or other members of the study team. Interviews will last about 30-45 minutes. Group interviews/talking circles will take place at a location within 20 minutes of Downtown Minneapolis (Phillips neighborhood) or in Rochester, MN OR within 20 minutes of Downtown/Central Phoenix, AZ. You will be asked questions about smoking including, but not limited to, why you have or have not quit, what triggers you to smoke, types of tobacco you use or currently use, if you use traditional tobacco, and what tools you have used or would like to use to quit smoking. You may also be asked questions about pregnancy and smoking if you are planning pregnancy or have been pregnant.

All participants who complete a group or 1:1 interview will be paid $50 in the form of a virtual cash card. Those who participate in the in-person group interviews be given a cultural gift (value of less than $10, and will have the option to receive nicotine replacement products such as nicotine gum or patches. You will be paid using the Greenphire ClinCard, a pre-paid debit card that functions like a bank debit card. The study team will load your study payments on the Greenphire ClinCard once you complete a study visit. For Mayo Clinic employees, research payments are included in your paycheck.

To get paid with the ClinCard, Greenphire will need to process certain personal information about you. Your name, address, and date of birth will be given to Greenphire ClinCard. This information will be collected from you by the study staff and given to Greenphire. If you choose to not provide the required information to Greenphire, the study team can have a check issued to you through the mail. However, your information will still be collected by the study team to issue the payment via check.

Payment for participation in research is considered taxable income and reportable to the Internal Revenue Service (IRS). If you receive research payments from Mayo Clinic totaling $600 or more in a calendar year, a tax Form 1099 will be sent to you.

This research study will not directly benefit you. The results will be used to create a smoking cessation intervention designed to help Native people quit smoking.

The risks associated with the research study may include discomfort or emotional stress with questions during the interview. You do not have to answer any question that makes you uncomfortable, or that you do not want to answer. Your information collected as a part of this research will not be used or distributed for future research. Your health information will not be accessed as part of this research study. Loss of confidentiality is a risk of this study, as with all research studies. All data (including interview recordings and transcriptions) will be stored on a secure server only accessible to approved study staff, and will be deleted after the completion of study.

Please understand your participation is voluntary and you have the right to withdraw your consent or discontinue participation at any time without penalty. Specifically, your current or future medical care, benefits, or employment at the Mayo Clinic will not be jeopardized if you choose not to participate. We recognize American Indian and Alaska Native data is protected and data is not owned by the research team. This research study was created with input and feedback from Minnesota and Arizona community advisory boards, and the Healthy Nations Advisory board with ongoing continuous feedback for the duration of the study.

If you have any questions about this research study, you can contact the study team at RuskSmokingStudy@mayo.edu. If you have any concerns, complaints, or general questions about research or your rights as a participant, please contact the Mayo Institutional Review Board (IRB) to speak to someone independent of the research team at 507-266-4000 or toll free at 866-273-4681.

If you are interested in participating in this study, please answer the question below and continue to the screening questionnaire.

Are you interested in participating in this study?

If no: Thank you for your time!

If yes: Continue to screening

Mayo Clinic: Office for Human Research Protection

**Oral Consent Script – American Indian and Alaska Native Subjects**

**Parents/Guardians of Minor Participants (age 14-17)**

**Protocol Title:** Understanding barriers to smoking cessation in American Indian and Alaska Native People

**IRB #:** 23-011852

**Principal Investigator:** Dr. Ann M. Rusk, M.D., and colleagues

Please read the oral consent script below to learn more about the study.

Your child is being asked to participate in a research study about the experience of quitting cigarette smoking. The purpose of this research is to understand barriers to long-term smoking cessation in American Indian or Alaska Native people and barriers to quitting during pregnancy. If your child self-identifies as American Indian or Alaska Native and they used to or currently smoke cigarettes, they may be eligible for this study.

If your child agrees to participate, they will be asked to do a 1:1 discussion with Dr. Rusk and/or other members of the study team. Interviews will be conducted via phone, or video call, and will last about 30-45 minutes. Your child will be asked questions about smoking including, but not limited to, why they have or have not quit, what triggers them to smoke, types of tobacco they use or currently use, if they use traditional tobacco, and what tools they have used or would like to use to quit smoking. They may also be asked questions about pregnancy and smoking if they are planning pregnancy or have been pregnant.

If your child completes the interview, they will be paid $50 in the form of a virtual cash card. They will be paid using the Greenphire ClinCard, a pre-paid debit card that functions like a bank debit card. The study team will load the study payment on the Greenphire ClinCard once they complete the interview.

Because your child is under 18 years of age, the remuneration will be paid to you as a parent or guardian, and your information will be collected to process the payment. To get paid with the ClinCard, Greenphire will need to process certain personal information about you. Your name, address, and date of birth will be given to Greenphire ClinCard. This information will be collected by the study staff and given to Greenphire. If you choose to not provide the required information to Greenphire, the study team can have a check issued to you through the mail. However, your information will still be collected by the study team to issue the payment via check.

Payment for participation in research is considered taxable income and reportable to the Internal Revenue Service (IRS). If you receive research payments from Mayo Clinic totaling $600 or more in a calendar year, a tax Form 1099 will be sent to you.

This research study will not directly benefit your child. The results will be used to create a smoking cessation intervention designed to help Native people quit smoking.

The risks associated with the research study may include discomfort or emotional stress with questions during the interview. Your child does not have to answer any question that makes them uncomfortable, or that they do not want to answer. Information collected as a part of this research will not be used or distributed for future research. Your child’s health information will not be accessed as part of this research study. Loss of confidentiality is a risk of this study, as with all research studies. All data (including interview recordings and transcriptions) will be stored on a secure server only accessible to approved study staff, and will be deleted after the completion of study.

Please understand your child’s participation is voluntary and they have the right to withdraw their consent or discontinue participation at any time without penalty. Specifically, their current or future medical care or benefits at the Mayo Clinic will not be jeopardized if they choose not to participate. We recognize American Indian and Alaska Native data is protected and data is not owned by the research team. This research study was created with input and feedback from Minnesota and Arizona community advisory boards, and the Healthy Nations Advisory board with ongoing continuous feedback for the duration of the study.

All data collected for this study is confidential and will not be released to parents or legal guardians. Your child’s pregnancy status will not be communicated to you without permission. But, if the Principal Investigator believes that being pregnant may cause serious health problems, they may need to tell you about your child’s pregnancy status.

If your child has any questions about this research study, they can contact the study team at [RuskSmokingStudy@mayo.edu](mailto:RuskSmokingStudy@mayo.edu). If you or your child have any concerns, complaints, or general questions about research or their rights as a participant, please contact the Mayo Institutional Review Board (IRB) to speak to someone independent of the research team at 507-266-4000 or toll free at 866-273-4681.

If your child is interested in participating in this study, please answer the question below and continue to the screening questionnaire.

For the participant (age 14-17): Are you interested in participating in this study?

If no: Thank you for your time!

If yes: Continue to parent permission

For the parent/guardian of the child participant: Do you give your permission to allow your child to participate in this study?

If no: Thank you for your time!

If yes/fill in parent/guardian name: Continue to screening

Mayo Clinic: Office for Human Research Protection

**Oral Consent Script – Smoking Cessation Counselors**

**Protocol Title**: Understanding barriers to smoking cessation in American Indian and Alaska Native People

**IRB #:** 23-011852

**Principal Investigator:** Dr. Ann M. Rusk, M.D., and colleagues

Please read the oral consent script below to learn more about the study.

You are being asked to participate in a research study addressing barriers to smoking cessation in American Indians and Alaska Natives. The purpose of this research is to understand barriers to long-term smoking cessation in American Indian or Alaska Native people and barriers to quitting during pregnancy. If your work includes counseling or advising patients who are American Indian or Alaska Native on ways to quit smoking, you may be eligible for this study.

If you agree to participate you will be asked to complete a recorded interview with the Principal Investigator, Dr. Ann Rusk and/or other members of the study team. The interview will include 3-4 people and will last about 30-45 minutes. Interviews will be conducted in-person at a location within 20 minutes of Downtown Minneapolis (Phillips neighborhood) or in Rochester, MN OR within 20 minutes of Downtown/Central Phoenix, AZ.. You will be asked questions about your work offering cessation counseling, treatments offered to your patients, and review items that will be considered for a future survey on treatment options.

If you complete the interview, you will be paid $50 in the form of a cash card, or if you are a Mayo employee, this payment will be added to your paycheck. You will be paid using the Greenphire ClinCard, a pre-paid debit card that functions like a bank debit card. The study team will load your study payments on the Greenphire ClinCard once you complete a study visit. For Mayo Clinic employees, research payments are included in your paycheck.

To get paid with the ClinCard, Greenphire will need to process certain personal information about you. Your name, address, and date of birth will be given to Greenphire ClinCard. This information will be collected from you by the study staff and given to Greenphire. If you choose to not provide the required information to Greenphire, the study team can have a check issued to you through the mail.

Payment for participation in research is considered taxable income and reportable to the Internal Revenue Service (IRS). If you receive research payments totaling $600 or more in a calendar year, a tax Form 1099 will be sent to you. For Mayo Clinic employees, research payments are included in your paycheck with applicable taxes withheld and reported on your Form W2 after calendar year-end.

This research study will not directly benefit you. The results will be used to create a smoking cessation intervention designed to help Native people quit smoking.

The risks associated with the research study may include discomfort or emotional stress with questions during the interview. You do not have to answer any question that makes you uncomfortable, or that you do not want to answer. Your information collected as a part of this research will not be used or distributed for future research. Your health information will not be accessed as part of this research study. Loss of confidentiality is a risk of this study, as with all research studies. All data (including interview recordings and transcriptions) will be stored on a secure server only accessible to approved study staff, and will be deleted after the completion of study.

Please understand your participation is voluntary and you have the right to withdraw your consent or discontinue participation at any time without penalty. Specifically, your current or future medical care or employment at the Mayo Clinic will not be jeopardized if you choose not to participate. We recognize American Indian and Alaska Native data is protected and data is not owned by the research team. This research study was created with input and feedback from Minnesota and Arizona community advisory boards, and the Healthy Nations Advisory board with ongoing continuous feedback for the duration of the study.

If you have any questions about this research study you can contact the study team at [RuskSmokingStudy@mayo.edu](mailto:RuskSmokingStudy@mayo.edu). If you have any concerns, complaints, or general questions about research or your rights as a participant, please contact the Mayo Institutional Review Board (IRB) to speak to someone independent of the research team at 507-266-4000 or toll free at 866-273-4681.

If you are interested in participating in this study, please answer the question below and continue to the screening questionnaire.

Are you interested in participating in this study?

If no: Thank you for your time!

If yes: Continue to screening
