# Supplementary material for: Identifying pragmatic solutions to reduce cigarette smoking prevalence in Indigenous North Americans: A sequential exploratory mixed-methods study protocol
Source: PLoS One. 2024 Nov 11;19(11):e0306512. doi: 10.1371/journal.pone.0306512 (PMC11554222; doi:10.1371/journal.pone.0306512)
Supplement: S2 Appendix — (DOCX) [file pone.0306512.s002.docx]

**S2 Appendix: Sample DCE Survey**

**Choice set for smoking relapse**

Imagine you are a former smoker who quit more than a year ago, and you just restarted smoking. You know there is a new, free program developed by your local healthcare office to help Native people remain smoke free after quitting, support people who have started smoking again. The program includes access to a counselor, options for quit aids if needed, and options for group support. Given the details between option A or option B, which would you pick?

| **Treatment Element** | **Option A** | **Option B** |
| --- | --- | --- |
| Individual meeting with smoking counselor | Over the phone | In person |
| Free nicotine replacement therapy (such as gum, lozenges, inhalers, or a patch) | Delivered by mail | Pick up at a clinic or health office |
| Connection with other people who have quit smoking | An option to join or view an online group on social media with former smokers (such as Facebook or Instagram) | An option for in-person meetings with other people in my community who are former smokers |
| Topics covered by a smoking counselor | I would like to hear about treatment options available | I would like to talk about why I am thinking of smoking again |
| Follow up with smoking counselors | I would like to have follow up scheduled right away | I would like to schedule appointments as needed |
| Free prescriptions or other drug therapies (such as bupropion, Wellbutrin, Zyban, Chantix, or varenicline) | Having easy access to prescriptions is important to help me quit again | It is not important to me to have access to prescription medications |
| Advertising for this program | Seeing what services are available with minimal extra information is important | In addition to services available to me, imagery and photos from my community are important |
| **I prefer the following option (check only one box)** | **Option A**  **☐** | **Option B**  **☐** |

**OR**

**☐** *I prefer neither of these options*

**Choice set for quitting during pregnancy**

Imagine you smoke cigarettes and are three months pregnant. You would like to quit smoking before your baby is born. You know there is a treatment plan being offered by your local clinic. Given the following choices between option A and option B, which plan would be more helpful to get you to quit smoking before the baby is born?

| **Treatment Element** | **Option A** | **Option B** |
| --- | --- | --- |
| Meeting with a smoking counselor | It is important the team managing my pregnancy is involved in my plan to quit smoking | I would like to talk to a counselor outside of the team taking care of my pregnancy |
| Topics discussed with a smoking counselor | Risks to me and my baby because of smoking | Discussing the reasons why I smoke |
| Frequency of meetings | I would like meetings with a smoking cessation counselor to be scheduled immediately after my prenatal visits | I would like to meet every week until I have reached my goal |
| Demographics of counselor | The age, race, and gender of my counselor is not a critical factor to me | I would like to talk to someone who has experienced pregnancy before |
| Connection to network of other people | I would like to have the option to access a website or social media page such as Facebook or Instagram including other people who have smoked during pregnancy | I would like the option to attend an in-person meeting with other people who have smoked during pregnancy |
| Advertising for this program | Seeing what services are available with minimal extra information is important | In addition to services available to me, imagery and photos from my community are important |
| **I prefer the following option (check only one box)** | **Option A**  **☐** | **Option B**  **☐** |

**OR**

**☐** *I prefer neither of these options*
