# Supplementary material for: Identifying pragmatic solutions to reduce cigarette smoking prevalence in Indigenous North Americans: A sequential exploratory mixed-methods study protocol
Source: PLoS One. 2024 Nov 11;19(11):e0306512. doi: 10.1371/journal.pone.0306512 (PMC11554222; doi:10.1371/journal.pone.0306512)
Supplement: Attachment — Submitted filename: Response to the editors PLOS ONE.docx [file pone.0306512.s003.docx]

To Hillary Genn Givero and the editors of PLOS ONE,

Thank you for your timely and thoughtful review of our manuscript. In addition to the tracked changes in attached manuscripts, please note the following responses for consideration of submission for review. Please let me know if there are additional questions, concerns, or comments and I am happy to follow up.

Best,

Annie Rusk

*(1) Please note that the Study Protocol article type is only suitable for proposals of studies that have not yet generated results. For further information, please see* [*https://nam12.safelinks.protection.outlook.com/?url=https%3A%2F%2Fjournals.plos.org%2Fplosone%2Fs%2Fsubmission-guidelines%23loc-study-protocols&data=05%7C02%7CRusk.Ann%40mayo.edu%7Ce772996b0ab0467bd0b408dc1db1a460%7Ca25fff9c3f634fb29a8ad9bdd0321f9a%7C0%7C0%7C638417896009072025%7CUnknown%7CTWFpbGZsb3d8eyJWIjoiMC4wLjAwMDAiLCJQIjoiV2luMzIiLCJBTiI6Ik1haWwiLCJXVCI6Mn0%3D%7C3000%7C%7C%7C&sdata=kglkX%2BrD6Bu4JgBHHhuAV4aNQMvSaQpjOrH1vIxQoXk%3D&reserved=0*](https://nam12.safelinks.protection.outlook.com/?url=https%3A%2F%2Fjournals.plos.org%2Fplosone%2Fs%2Fsubmission-guidelines%23loc-study-protocols&data=05%7C02%7CRusk.Ann%40mayo.edu%7Ce772996b0ab0467bd0b408dc1db1a460%7Ca25fff9c3f634fb29a8ad9bdd0321f9a%7C0%7C0%7C638417896009072025%7CUnknown%7CTWFpbGZsb3d8eyJWIjoiMC4wLjAwMDAiLCJQIjoiV2luMzIiLCJBTiI6Ik1haWwiLCJXVCI6Mn0%3D%7C3000%7C%7C%7C&sdata=kglkX%2BrD6Bu4JgBHHhuAV4aNQMvSaQpjOrH1vIxQoXk%3D&reserved=0)

*Please update your Cover Letter to indicate whether you wish to have the manuscript considered as a Study Protocol, and confirm that neither recruitment nor data collection have been completed.*

**The cover letter has been updated to indicate that as of today’s date, 1/25/2024, subject recruitment is not complete and no data collection has begun. Please see tracked changes in the cover letter as well.**

(2) Please report in the Methods section the tentative day, month and year of the start and end of the recruitment period for this study.

**Recruitment beginning with a goal of January 2024 and conclusion by end of 2024 have been added to the methods section, page 9, in the tracked changes.**

(3) We have noted that you state "To protect the privacy of pediatric patients, those under age 18 who have parental consent and who provide assent to participate will only be eligible for arm 1". However, you also mention that minors (age 14-17) will be interviewed as part of arm 2." Could you please update the methodology to ensure that it is consistent?

**Thank you for highlighting confusing terminology, this has been corrected in the methods, with tracked changes noted on page 9.**

(4) Please, could you also upload a copy of the interview guide that you will use for the semi-structured interviews in arm 2?

**Interview guide with IRB approval denoted has been uploaded.**
