# Supplementary material for: Identifying pragmatic solutions to reduce cigarette smoking prevalence in Indigenous North Americans: A sequential exploratory mixed-methods study protocol
Source: PLoS One. 2024 Nov 11;19(11):e0306512. doi: 10.1371/journal.pone.0306512 (PMC11554222; doi:10.1371/journal.pone.0306512)
Supplement: Attachment — Submitted filename: Revision 1 Response to Reviewers.docx [file pone.0306512.s004.docx]

Response to Reviewers:

4/1/2024

The authors would like to thank the PLOS ONE editorial team and reviewers for the thoughtful and detailed review of this protocol paper. We have conducted edits as highlighted below, and there are responses in two sections below, first, in “Journal Requirements” and second, in “Reviewers’ Comments.” Please note my comments are italicized in sections requiring a response for ease of reading. Please do not hesitate to contact me regarding additional comments, questions, or concerns regarding this manuscript.

Best,

Annie Rusk

**Journal Requirements:**

When submitting your revision, we need you to address these additional requirements.

1. Please ensure that your manuscript meets PLOS ONE's style requirements, including those for file naming. The PLOS ONE style templates can be found at

<https://journals.plos.org/plosone/s/file?id=wjVg/PLOSOne_formatting_sample_main_body.pdf> and

*The headings, level 2 and level 3 headings have been modified to match formatting requirements. Bold had been removed from figure labels. See tracked changes.*

<https://journals.plos.org/plosone/s/file?id=ba62/PLOSOne_formatting_sample_title_authors_affiliations.pdf>

*Title page and author affiliations have been changed to match journal formatting requirements. See tracked changes.*

2. Please include a complete copy of PLOS’ questionnaire on inclusivity in global research in your revised manuscript. Our policy for research in this area aims to improve transparency in the reporting of research performed outside of researchers’ own country or community. The policy applies to researchers who have travelled to a different country to conduct research, research with Indigenous populations or their lands, and research on cultural artefacts. The questionnaire can also be requested at the journal’s discretion for any other submissions, even if these conditions are not met. Please find more information on the policy and a link to download a blank copy of the questionnaire here: <https://journals.plos.org/plosone/s/best-practices-in-research-reporting>. Please upload a completed version of your questionnaire as Supporting Information when you resubmit your manuscript.

*Please see completed inclusivity in global research questionnaire in submitted documents.*

3. In the ethics statement in the Methods, you have specified that verbal consent was obtained. Please provide additional details regarding how this consent was documented and witnessed, and state whether this was approved by the IRB.

*The authors appreciate this comment, and have clarified the methods of consent, which can now be found under subheading “consent” following the “Indigenous Research Considerations and Community Engagement Plan”. Consent strategies are described, which include review of verbal and electronic scripts, as well as repeat of information immediately before interviews. Survey consent may occur in multiple methods, which has been highlighted in the methods section. The IRB approved materials have also been included as “Appendix 1”.*

4. We note that the grant information you provided in the ‘Funding Information’ and ‘Financial Disclosure’ sections do not match.

When you resubmit, please ensure that you provide the correct grant numbers for the awards you received for your study in the ‘Funding Information’ section.

*Please see updated funding information in manuscript and disclosures.*

5. Thank you for stating the following financial disclosure:

“Robert D. and Patricia E. Kern Center for the Science of Healthcare Delivery, Robert A. Winn Career Development Award, American Thoracic Society Fellowship in Health Equity and Diversity.”

Please state what role the funders took in the study.  If the funders had no role, please state: "The funders had no role in study design, data collection and analysis, decision to publish, or preparation of the manuscript."

If this statement is not correct you must amend it as needed.

Please include this amended Role of Funder statement in your cover letter; we will change the online submission form on your behalf.

*The role of funder did not include design, data collection or analysis, publication, or manuscript preparation or production, and this statement has been added to the cover letter.*

6. Thank you for stating the following in the Acknowledgments Section of your manuscript:

“The authors would like to acknowledge and thank the generous funders supporting this study, including the Robert D. and Patricia E. Kern Center for the Science of Healthcare Delivery; The American Thoracic Society; The Robert A. Winn Career Development Award; the Mayo Clinic Center for Clinical and Translational Science. The authors would also like to thank the ongoing input and participation from community members who have participated in Community Engagement Studios, advice from the Healthy Nations Advisory Board, and the input from the Mayo Clinic Centers for Health Equity and Community Engagement Research in Rochester, MN, and Phoenix, AZ, for the support of this study.”

We note that you have provided funding information that is currently declared in your Funding Statement. However, funding information should not appear in the Acknowledgments section or other areas of your manuscript. We will only publish funding information present in the Funding Statement section of the online submission form.

*The authors thank the reviewers for clarifying where funding information should appear. The acknowledgement section has been adjusted accordingly, please see tracked changes for updates and details.*

Please remove any funding-related text from the manuscript and let us know how you would like to update your Funding Statement. Currently, your Funding Statement reads as follows:

“Robert D. and Patricia E. Kern Center for the Science of Healthcare Delivery, Robert A. Winn Career Development Award, American Thoracic Society Fellowship in Health Equity and Diversity.”

*Funding information has been removed from the manuscript, the above statement is correct.*

Please include your amended statements within your cover letter; we will change the online submission form on your behalf.

*See updated cover letter, thank you for clarification.*

7. Your ethics statement should only appear in the Methods section of your manuscript. If your ethics statement is written in any section besides the Methods, please delete it from any other section.

*Sentence regarding ethics has been removed from “Conclusions” section, see tracked changes.*

8. Please include a separate caption for each figure in your manuscript.

*Figures have been updated to match above guidelines and descriptions/captions added to figure files.*

9. Please include captions for your Supporting Information files at the end of your manuscript, and update any in-text citations to match accordingly. Please see our Supporting Information guidelines for more information: <http://journals.plos.org/plosone/s/supporting-information>.

*The appendices have been renamed in the text and in file nomenclature to match the requirements of the journal.*

10. Please review your reference list to ensure that it is complete and correct. If you have cited papers that have been retracted, please include the rationale for doing so in the manuscript text, or remove these references and replace them with relevant current references. Any changes to the reference list should be mentioned in the rebuttal letter that accompanies your revised manuscript. If you need to cite a retracted article, indicate the article’s retracted status in the References list and also include a citation and full reference for the retraction notice.

*I have reviewed citations with tools from my citation manager software and have not identified any retractions.*

Reviewers’ Comments

1. Does the manuscript provide a valid rationale for the proposed study, with clearly identified and justified research questions?

The research question outlined is expected to address a valid academic problem or topic and contribute to the base of knowledge in the field.

Reviewer #1: Yes

Reviewer #2: Yes

2. Is the protocol technically sound and planned in a manner that will lead to a meaningful outcome and allow testing the stated hypotheses?

The manuscript should describe the methods in sufficient detail to prevent undisclosed flexibility in the experimental procedure or analysis pipeline, including sufficient outcome-neutral conditions (e.g. necessary controls, absence of floor or ceiling effects) to test the proposed hypotheses and a statistical power analysis where applicable. As there may be aspects of the methodology and analysis which can only be refined once the work is undertaken, authors should outline potential assumptions and explicitly describe what aspects of the proposed analyses, if any, are exploratory.

Reviewer #1: Yes

Reviewer #2: Yes

3. Is the methodology feasible and described in sufficient detail to allow the work to be replicable?


Descriptions of methods and materials in the protocol should be reported in sufficient detail for another researcher to reproduce all experiments and analyses. The protocol should describe the appropriate controls, sample size calculations, and replication needed to ensure that the data are robust and reproducible.

Reviewer #1: Yes

Reviewer #2: Yes

4. Have the authors described where all data underlying the findings will be made available when the study is complete?

The [PLOS Data policy](https://nam12.safelinks.protection.outlook.com/?url=https%3A%2F%2Fjournals.plos.org%2Fplosone%2Fs%2Fmaterials-and-software-sharing&data=05%7C02%7CRusk.Ann%40mayo.edu%7C6aed1b9d24d7466c293408dc3899631f%7Ca25fff9c3f634fb29a8ad9bdd0321f9a%7C0%7C0%7C638447479251355901%7CUnknown%7CTWFpbGZsb3d8eyJWIjoiMC4wLjAwMDAiLCJQIjoiV2luMzIiLCJBTiI6Ik1haWwiLCJXVCI6Mn0%3D%7C0%7C%7C%7C&sdata=r1dPzQfby7MK63V3KyGhNKp3JjxZxeUeIAbxY2aVKLQ%3D&reserved=0) requires authors to make all data underlying the findings described in their manuscript fully available without restriction, with rare exception, at the time of publication. The data should be provided as part of the manuscript or its supporting information, or deposited to a public repository. For example, in addition to summary statistics, the data points behind means, medians and variance measures should be available. If there are restrictions on publicly sharing data—e.g. participant privacy or use of data from a third party—those must be specified.

Reviewer #1: Yes

Reviewer #2: Yes

5. Is the manuscript presented in an intelligible fashion and written in standard English?

PLOS ONE does not copyedit accepted manuscripts, so the language in submitted articles must be clear, correct, and unambiguous. Any typographical or grammatical errors should be corrected at revision, so please note any specific errors here.

Reviewer #1: Yes

Reviewer #2: Yes

6. Review Comments to the Author

Please use the space provided to explain your answers to the questions above and, if applicable, provide comments about issues authors must address before this protocol can be accepted for publication. You may also include additional comments for the author, including concerns about research or publication ethics.

You may also provide optional suggestions and comments to authors that they might find helpful in planning their study.

(Please upload your review as an attachment if it exceeds 20,000 characters)

Reviewer #1: The mixed-methods approach's comprehensive nature is indeed a valid point; integrating both qualitative and quantitative research provides a richer, more nuanced understanding of the targeted behaviors and experiences. The paper rightly suggests that this methodological synergy can enhance the depth and breadth of the findings, offering a more holistic perspective that pure quantitative or qualitative studies might not achieve. However, the argument's strength is directly related to the implementation quality. Simply having a mixed-methods study does not automatically translate to comprehensive insights. The concrete integration of these methods, data triangulation, and how the results will complement each other to inform the intervention are critical considerations.
The paper should clarify how the discrete choice experiments are designed, ensuring their scenarios rightly reflect realistic smoking cessation options, and that participants understand and can relate to the choices presented. The validity of the method hinges upon the extent to which these experiments mimic real-life decision-making contexts. Without this assurance, there's a valid point about the potential for compromised validity of the findings.
The localization of the study in Olmsted County, Minnesota, does raise concerns about how well the results can be extrapolated to AI/AN populations elsewhere. This limitation is important, as it affects the external validity and applicability of the results
While the paper may not address the long-term sustainability of the interventions developed, this presents a valid criticism that future research should consider. Sustainability is crucial for the long-term success of any health intervention, and the paper could improve by discussing potential strategies to maintain and scale the interventions beyond the study's scope.
Overall, the paper's mixed-methods approach, community engagement, focus on relapse prevention, and smoking during pregnancy provide strong arguments for the development of effective smoking cessation interventions in AI/AN communities. However, concerns regarding the sample size, validity of discrete choice experiments, generalizability, ethical considerations, and sustainability require further elaboration to establish the full strength and applicability of these arguments.

*The authors acknowledge the challenges of implementing interventions in diverse systems with variable resources. These limitations may be profound, and edits have been added in the tracked changes of the document in the discussion. Further description of validation of the DCE survey has also been added to the methods. The authors appreciate this thoughtful comment highlighting the importance of acknowledging the diversity that exists among AI/AN people in the US.*

Reviewer #2: Overall, this is a clear, and well-written manuscript. The introduction is relevant. This is an interesting study and the data will be informative. The study design is interesting. The methodology is feasible. The manuscript is structured. It is appropriate in length.

7. PLOS authors have the option to publish the peer review history of their article ([what does this mean?](https://nam12.safelinks.protection.outlook.com/?url=https%3A%2F%2Fjournals.plos.org%2Fplosone%2Fs%2Feditorial-and-peer-review-process%23loc-peer-review-history&data=05%7C02%7CRusk.Ann%40mayo.edu%7C6aed1b9d24d7466c293408dc3899631f%7Ca25fff9c3f634fb29a8ad9bdd0321f9a%7C0%7C0%7C638447479251362019%7CUnknown%7CTWFpbGZsb3d8eyJWIjoiMC4wLjAwMDAiLCJQIjoiV2luMzIiLCJBTiI6Ik1haWwiLCJXVCI6Mn0%3D%7C0%7C%7C%7C&sdata=555pvVMzn4N58BAYZ9d232IB%2BgbxoL%2F3ZEy6tT2Qico%3D&reserved=0)). If published, this will include your full peer review and any attached files.


If you choose “no”, your identity will remain anonymous but your review may still be made public.


**Do you want your identity to be public for this peer review?** For information about this choice, including consent withdrawal, please see our [Privacy Policy](https://nam12.safelinks.protection.outlook.com/?url=https%3A%2F%2Fwww.plos.org%2Fprivacy-policy&data=05%7C02%7CRusk.Ann%40mayo.edu%7C6aed1b9d24d7466c293408dc3899631f%7Ca25fff9c3f634fb29a8ad9bdd0321f9a%7C0%7C0%7C638447479251368399%7CUnknown%7CTWFpbGZsb3d8eyJWIjoiMC4wLjAwMDAiLCJQIjoiV2luMzIiLCJBTiI6Ik1haWwiLCJXVCI6Mn0%3D%7C0%7C%7C%7C&sdata=KklklNHEUq6np%2Byh4LxY996QgC4h9eFx8WPHLVOo%2BEw%3D&reserved=0).

Reviewer #1: No

Reviewer #2: No
